# Revealing Further Insights into Astringent Seeds of Chinese Fir by Integrated Metabolomic and Lipidomic Analyses

**DOI:** 10.3390/ijms242015103

**Published:** 2023-10-12

**Authors:** Ping Zheng, Mengqian Shen, Ruoyu Liu, Xinkai Cai, Jinting Lin, Lulu Wang, Yu Chen, Guangwei Chen, Shijiang Cao, Yuan Qin

**Affiliations:** 1 Fujian Provincial Key Laboratory of Haixia Applied Plant Systems Biology, Center for Genomics and Biotechnology, College of Life Science, Fujian Agriculture and Forestry University, Fuzhou 350002, China; zhengping13@mails.ucas.ac.cn (P.Z.); m_anonek@163.com (M.S.); caixinkai2003@163.com (X.C.); 3205410046@stu.fafu.edu.cn (J.L.); cgw316458611@gmail.com (G.C.); 2 Pingtan Science and Technology Research Institute, College of Marine Sciences, Fujian Agriculture and Forestry University, Fuzhou 350002, China; liuruoyu13@mails.ucas.ac.cn; 3 College of Forestry, Fujian Agriculture and Forestry University, Fuzhou 350002, China; 000q813024@fafu.edu.cn

**Keywords:** Chinese fir, astringent seeds, seed abortion, lipidome, metabolome

## Abstract

Chinese fir (*Cunninghamia lanceolata* (Lamb.) Hook.) stands as one of the pivotal afforestation tree species and timber resources in southern China. Nevertheless, the occurrence of seed abortion and a notably high proportion of astringent seeds significantly curtail the yield and quality of elite seeds, resulting in substantial economic losses. The development of astringent seeds is accompanied by significant physiological and biochemical alterations. Here, the first combined lipidomic and metabolomic analysis was performed to gain a comprehensive understanding of astringent seed traits. A total of 744 metabolites and 616 lipids were detected, of which 489 differential metabolites and 101 differential lipids were identified. In astringent seeds, most flavonoids and tannins, as well as proline and γ-aminobutyric acid, were more accumulated, along with a notable decrease in lipid unsaturation, indicating oxidative stress in the cells of astringent seeds. Conversely, numerous elemental metabolites were less accumulated, including amino acids and their derivatives, saccharides and alcohols, organic acids and nucleotides and their derivatives. Meanwhile, most lipid subclasses, mainly associated with energy storage (triglyceride and diglyceride) and cell membrane composition (phosphatidic acid, phosphatidylcholine, phosphatidylethanolamine), also exhibited significant reductions. These results reflected a disruption in the cellular system or the occurrence of cell death, causing a reduction in viable cells within astringent seeds. Furthermore, only one lipid subclass, sphingosine phosphate (SoP), was more accumulated in astringent seeds. Additionally, lower accumulation of indole-3-acetic acid and more accumulation of salicylic acid (SA) were also identified in astringent seeds. Both SA and SoP were closely associated with the promotion of programmed cell death in astringent seeds. Collectively, our study revealed significant abnormal changes in phytohormones, lipids and various metabolites in astringent seeds, allowing us to propose a model for the development of astringent seeds in Chinese fir based on existing research and our findings. This work enriches our comprehension of astringent seeds and presents valuable bioindicators for the identification of astringent seeds.

## 1. Introduction

Chinese fir (*Cunninghamia lanceolata* (Lamb.) Hook.) is an important timber species in southern China with a wide distribution and a long history of cultivation, holding significant economic value [1,2]. However, Chinese fir is exhibiting growth degeneration, with a significant occurrence of seed abortion in natural forests. Generally, the seed setting rate of Chinese fir cones is about 4–5%, with only 30–50% being intact seeds, while the rest are abnormal empty and astringent seeds [3]. Particularly, the proportion of astringent seeds is notably high and ranges from 50% to 60%. Even in seed orchards, the astringent seed rate can reach around 30% [4], severely impacting seed yield and quality, thereby constraining the development of Chinese fir breeding and causing substantial economic losses.

Over the past decades, extensive investigations have been conducted on the issue of astringent seeds in Chinese fir, including anatomical analysis [2,5] and identification of intrinsic components [6], physiological and biochemical changes [5,7,8], as well as ecological factors contributing to the production of astringent seeds [4,9,10]. Generally, the process of astringent seed formation can be divided into two stages: embryo abortion and astringent substance accumulation [11]. The process of astringent seed abortion seems to be closely related to the pollination process. In Chinese fir, after pollination, pollen initially remains at the micropylar end of the ovule. Following a period of dormancy, the pollen starts to germinate and extends towards the chalazal end. Similarly, in astringent seeds, cell death and disintegration, as well as the appearance of astringent substances, also first occur at the micropylar end of abortive ovules, gradually extending towards the chalazal end of the ovule [2]. Indeed, self-pollination is one of the primary factors leading to the formation of astringent seeds. The influence of difference of genetic traits on astringent seed formation might be linked to the occurrence of highly homozygous states due to inbreeding or self-pollination in certain genotypes [1,12]. In addition, adverse climate conditions, such as high temperatures, abundant rainfall, humidity and droughts, might disrupt the delicate processes of seed development and cause higher proportions of astringent seeds [10]. Furthermore, phytohormone or nutrient imbalances are also involved in the development of astringent seeds. During the development of astringent seeds, the levels of indole-3-acetic acid (IAA) and gibberellins (GA) gradually decrease, while abscisic acid (ABA) increases and becomes the dominant phytohormone. The imbalance of phytohormones in astringent seeds might lead to a decrease in their competitiveness and impact nutrient absorption; thus, inadequate nutrient availability further causes decreases in physiological activity in astringent seeds [4].

Astringent seeds represent an abortive phenomenon observed across many conifers, including *Pinus tabuliformis*, *Araucaria cunninghamii* and *Cedrus* spp. [13]. Existing investigations have shown that seed abortion involves multiple cellular processes such as elevated ROS production [14], programmed cell death (PCD) [15], membrane lipid remodeling [16], hormone signaling [17], nutrient transport [18] and some stress-response-related pathways [19]. In Chinese fir, the abortion of astringent seeds may be attributed to the damaging effect of reactive oxygen species (ROS) on embryo and endosperm cells, resulting in severe membrane damage and cell disintegration. The primary cause of this phenomenon is the reduced activity of the endogenous protective enzyme system (peroxidase, polyphenol oxidase, superoxide dismutase, etc.) within the astringent seed, leading to elevated accumulation of ROS within the cells [5]. The process of astringent seed development in Chinese fir is accompanied by significant physiological and biochemical changes. The common metabolic characteristics of astringent seeds in Chinese fir mainly include a notable increase in polyphenol metabolites such as flavonoids and tannins, a significant decrease in organic macromolecule contents such as proteins, nucleic acids, carbohydrates and lipids [10,20], a notable reduction in the soluble sugars and free amino acids [7] and unusual alterations of phytohormones [4,8]. Due to the slow growth rate and long seed maturation period of Chinese fir, current research on astringent seeds primarily relies on samples collected from the wild. Therefore, the exact causes of astringent seed formation cannot be attributed to a single factor. However, different studies have shown many similarities in the physiological and biochemical changes in astringent seeds [4,7,8,10], implying that various factors influencing astringent seed formation may share some common underlying mechanisms which need further investigation.

Metabolomics serves as an essential and effective tool to investigate the accumulation patterns of metabolites and their underlying metabolic foundations, providing insights into the physiological states of plant development or stress response at the metabolic level [21,22], such as pollen abortion in tomatoes [23] and astringent seed formation in Chinese fir [8]. Among these, lipidomics, as an important branch of metabolomics, aims to systematically study the lipids within organisms at a systemic level to unveil the pivotal mechanistic roles of lipids across a range of life activities [24]. Lipids are widely distributed metabolic products in plants and participate in various essential life processes [8,25,26]. Integrating lipidomics with other omics studies can complement the omics-derived knowledge and enable the development of mechanism models for the system as a whole. Currently, combined metabolic and lipidomic analyses have been applied to various plants, such as tobacco, rice, wheat, and cotton, contributing to more comprehensive insights into various aspects of plant growth/development and stress responses [27,28,29,30,31].

Lipids are crucial for energy storage, membrane formation and signaling processes during seed development, and proper lipid metabolism and composition are crucial for embryo growth and viability [25,32]. Previous studies have revealed that aberrant lipid alterations occurred in astringent seeds in Chinese fir [5,10]. Our preliminary observations of different seed morphologies highlighted the presence of a distinct oily droplet-like substance upon applying gentle pressure with tweezers, indicating a divergent lipid metabolism in astringent seeds. Despite some studies having explored the metabolic characteristics of astringent seeds, the changes in lipid metabolism remain unclear. This study employs comparative metabolomics and lipidomics analyses of astringent and normal seeds, contributing to a more comprehensive understanding of astringent seed traits and offering valuable indicators for enhancing the capability of identifying astringent seeds in Chinese fir.

## 2. Results

### 2.1. Sample Collection and Metabolite/Lipid Identification

**Figure 1:**
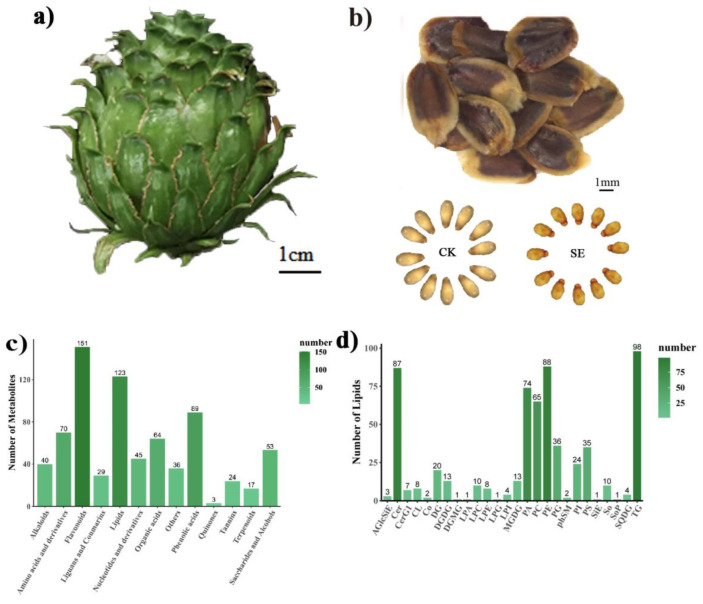
Sample collection and metabolite/lipid identification. (**a**) The cones of Chinese fir. (**b**) The seeds extracting from the cones and the morphological differences between astringent seeds (SE) and normal seeds (CK) after removing the seed coat. (**c**) Statistics of metabolites detected in different categories. (**d**) Statistics of lipid species detected in different lipid subclasses. The abbreviations for lipid subclasses are listed in Table 1.

After extracting all seeds from the collected cones (Figure 1a) and excluding empty seeds, it was challenging to visually distinguish between astringent and normal seeds based on their external morphology. After removing the seed coat, it was observed that the surface of the embryos of astringent seeds exhibited a light brown-red color, while the embryos of normal seeds were creamy-white (Figure 1b). An untargeted metabolomics analysis was performed to profile the metabolic changes in astringent seeds; normal seeds were used as control. In the metabolomic analysis, a total of 744 metabolites were identified, including 151 flavonoids, 123 lipids, 89 phenolic acids, 70 amino acids and their derivatives, 64 organic acids, 53 saccharides and alcohols, 45 nucleosides and their derivatives, 40 alkaloids, 29 lignans and coumarins, 24 tannins, 17 terpenoids and 36 other metabolites such as vitamins, stilbene and xanthone (Figure 1c, Table S1). Among all the identified metabolites, flavonoids were found to be the most abundant class, followed by lipids and phenolic acids.

Apart from these previously reported phenotypic changes, we noticed distinct oily droplet-like substances in the areas where astringent seeds were gently pressed with forceps under a microscope. This phenomenon was consistently observed across more than a dozen randomly selected astringent seeds, indicating a potential abnormality in the lipid metabolism of astringent seeds. Thus, a lipidomic analysis was further conducted to investigate the lipid alterations in astringent seeds compared with normal seeds. Lipids are generally categorized into three classes: major types, subclasses and individual lipid species [33]. Here, 26 lipid classes and 616 lipid species belonging to six major types were identified across all samples (Table 1, Figure 1d), of which 57.5% (354) were glycerophospholipids, 19.2% (118) were glycerides, 16.2% (100) were sphingolipids, 5.0% (31) were glycolipids, 0.6% (4) were steroids and 0.3% (2) were pregnenolones (Table S2). As for different lipid subclasses, triglycerides (TGs) were the most abundant lipid subclass, containing 98 lipid species, followed by phosphatidylethanolamines (PE, 88), ceramides (Cer, 87), phosphatidic acids (PA, 74), phosphatidylcholines (PC, 65) and others (Figure 1d).

### 2.2. Global Metabolic Alterations in Astringent Seeds

**Figure 2:**
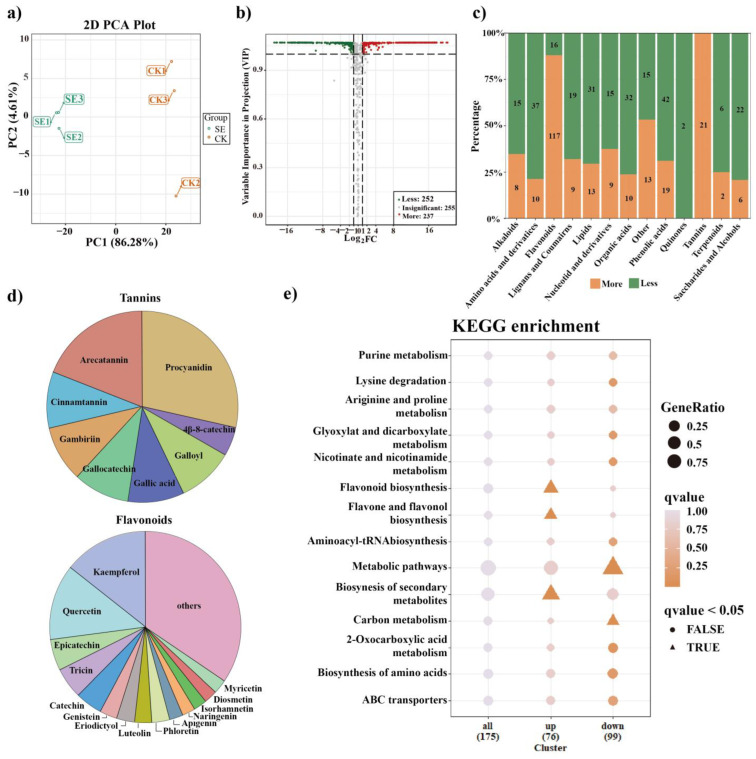
Metabolic alteration analysis in astringent seeds compared with normal seeds. (**a**) Principal component analysis (PCA); samples are grouped by different colors. “SE” indicates astringent seeds, “CK” indicates normal seeds. (**b**) Differentially accumulated metabolites (DAMs) shown in a volcano plot (fold change >2 or <0.5 and a *p* value less than 0.05). The more and less accumulated DAMs are highlighted in red and green, respectively. (**c**) The number of more and less accumulated DAMs in each category. (**d**) The proportions of subclasses in the two major categories of flavonoids and tannins. (**e**) KEGG enrichment analysis of all more accumulated and less accumulated DAMs.

Significantly differentially accumulated metabolites (DAMs) were screened out to explore the metabolic alterations in astringent seeds (SE) compared with normal seeds (CK). As shown in Figure 2a, a satisfactory explanation power for the control and sample groups was demonstrated by the PCA-X model, indicating that seed development and abortion processes of Chinese fir induced a clear metabolome perturbation. DAMs that led to the separation of the two groups are displayed in a volcano plot with following criteria: abs (log2(fold change)) > 1 and *p* values less than 0.05 (Figure 2b). A total of 489 DAMs were detected, of which 252 were less accumulated and 237 were more accumulated, including 133 flavonoids, 61 phenolic acids, 47 amino acids and their derivatives, 44 lipids, 42 organic acids, 28 saccharides and alcohols, 28 lignans and coumarins, 24 nucleosides and their derivatives, 23 alkaloids, 21 tannins, 8 terpenoids and 28 other metabolites (Table S1). The numbers of more and less accumulated DAMs in each category were counted separately and are shown in Figure 2c. Among them, 21 out of 24 DAMs in the tannin category (accounting for about 87.5%) were found to be more accumulated in astringent seeds, among which procyanidin and arecatannin were dominant (Figure 2d). Similarly, most DAMs in the flavonoid category (117 of 151, about 88.1%) were also more accumulated in astringent seeds, mainly including quercetin, kaempferol, epicatechin, tricin, catechin and others (Figure 2d). In addition, the top 15 more accumulated DAMs in astringent seeds all belonged to the categories of tannins (7), flavonoids (6) and phenolic acids (2) such as gallocatechin-catechin-catechin, procyanidin C1, sieboldin and arecatannin C1 (Table 2). These results revealed a significant increase in the accumulation of flavonoids and tannins in astringent seeds.

In contrast, over sixty percent of DAMs in other categories, including amino acids and their derivatives, organic acids, lipids, saccharides and alcohols, nucleotides and their derivatives and alkaloids, showed a decreased accumulation trend in astringent seeds (Figure 2c). Specifically, 22 out of 28 saccharide and alcohol DAMs were less accumulated in astringent seeds, including D-sucrose, D-maltose, D-panose, D-melezitose, nystose, stachyose, D-maltotetraose, raffinose, solatriose, isomaltulose, melibiose, D-trehalose and lactobiose (Table S1). As for organic acids, 32 DAMs were less accumulated in astringent seeds, including citric acid, isocitric acid, fumaric acid, succinic acid and L-malic acid, while only 10 DAMs were more accumulated, including shikimic acid, quinic acid, γ-aminobutyric acid (GABA), cis-aconitic acid and trans-citridic acid (Table S1). With regard to amino acids and their derivatives, 37 DAMs, including L-arginine, L-lysine, L-tryptophan, L-histidine and L-methionine, were less accumulated in astringent seeds, while only 10 DAMs were more accumulated, such as L-proline, L-asparagine, L-serine and L-ornithine (Table S1). In addition, two phytohormones were found to be significantly differentially accumulated between two groups, with indole 3-acetic acid (IAA, Log2FC = −13.34) in the alkaloid category being less accumulated, while salicylic acid (SA, Log2FC = 12.79) in the phenolic acid category being more accumulated in astringent seeds (Table S1). These findings shed light on the distinct characteristics and potential implications of these metabolites in relation to the observed variations.

To gain more insights into the specific metabolic processes, the DAMs were annotated and visualized using the Kyoto Encyclopedia of Genes and Genomes (KEGG) database (Figure 2e). The results showed that more accumulated DAMs were mainly enriched in “flavonoid biosynthesis”, “flavonoid and flavonol biosynthesis” and “biosynthesis of secondary metabolites”, while less accumulated DAMs were mainly enriched in “metabolic pathways” and “carbon metabolism”. These findings highlight the importance of these pathways in driving the observed metabolic variations.

### 2.3. Global Lipidomic Alterations in Astringent Seeds

Generally, lipid species within the same subclass share structural similarities and often exhibit similar biological functions. Consequently, lipid functions are mainly currently studied at the subclass level. Among the 26 detected lipid subclasses, 6 subclasses, namely digalactosylmonoacylglycerol (DGMG), lyso-phosphatidic acid (LPA), lyso-phosphatidylglycerol (LPG), lyso-phosphatidylinositol (LPI), sphingosine (So) and ceramides (Cer), did not show significant differences in accumulation between astringent seeds and normal seeds (Table 1). Only sphingosine phosphate (SoP), which belongs to the sphingolipids class, was significantly increased in astringent seeds, while the other 19 subclasses were significantly reducedin astringent seeds. These less accumulated lipid subclasses mainly included glycerolipids such as triacylglycerol (TG) and diacylglycerol (DG), as well as glycerophospholipids, encompassing phosphatidic acid (PA), phosphatidylcholine (PC), phosphatidylethanolamine (PE), etc. These observations collectively indicate substantial lipid metabolism alterations in astringent seeds compared with normal seeds.

**Figure 3:**
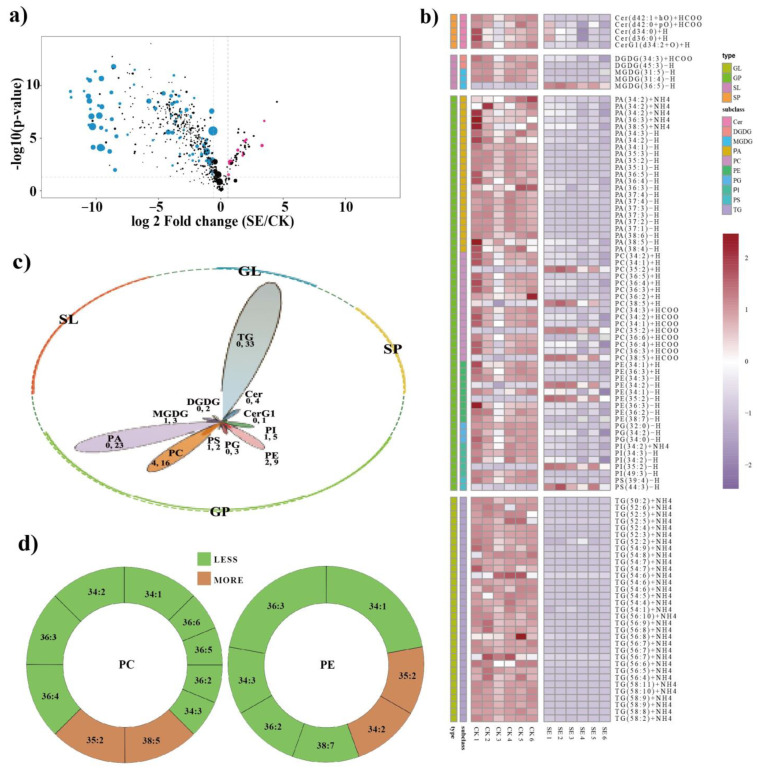
Lipidomic alteration analysis in astringent seeds compared with normal seeds. (**a**) Volcano plot analysis of differentially accumulated lipids (DALs). The more and less accumulated DALs are highlighted in rose-red and blue, respectively. (**b**) Heatmaps of DALs among the astringent seeds (SE) and normal seeds (CK). Differences in lipid accumulation changes are shown in color as the scale; red for more accumulation and blue for less accumulation. (**c**) Plot illustrating the number of more (before the comma) and less (after the comma) accumulated DALs in each subclass. (**d**) Circular plot illustrating the alterations of saturated and unsaturated fatty acids within the PC and PE subclasses. Brown and green, respectively, indicate more and less accumulated lipids.

For further lipid alteration assessment, lipids with a VIP > 1, a fold change > 1.5 or < 0.67, and a *p* value < 0.05 were designated as significantly differentially accumulated lipids (DALs). As depicted in the volcano plot (Figure 3a), a total of 101 DALs were identified between the two groups (Table S2). These DALs were predominantly found within 11 major lipid subclasses: TG (33), PA (23), PC (16), PE (9), phosphatidylinositol (PI, 5), Cer (4), phosphatidylglycerol (PG, 3), monogalactosyldiacylglycerol (MGDG, 3), phosphatidylserine (PS, 2), digalactosyldiacylglycerol (DGDG, 2) and glucosylceramide1 (CerG1, 1) (Figure 3b). The majority of these DALs were sourced from four main lipid types: glycolipids (TG and DG), glycerophospholipids (PA, PC, PE, PI, PG, PS, etc.), sphingolipids (Cer, CerG1, SoP, etc.) and glyceroglycolipids (MGDG and DGDG). Notably, four lipid subclasses—TG, PA, PC and PE—accounted for a substantial proportion (Figure 3b). Among all detected DALs between the two groups, 92 out of 101 DALs were less accumulated, of which 94.6% DALs (87 of 92) were unsaturated lipids (Table S2), highlighting a notable reduction in unsaturated fatty acid content in astringent seeds. Taking the changes in PC and PE subclasses as examples, the reduction in unsaturated fatty acids contributed significantly; PE (36:3), PE (34:1), PC (36:4), PC (36:3), PC (34:2) and PC (34:1) exhibited obviously decreasing trends (Figure 3c).

## 3. Discussion

Astringent seeds represent an abortive phenomenon observed in Chinese fir, which significantly undermines the production of high-quality seeds. Extensive investigations have been conducted on astringent seeds in Chinese fir, revealing that the process of astringent seed development is accompanied by significant physiological and biochemical changes. However, the relationship between metabolism alteration and seed embryo abortion in astringent seeds is not yet fully understood, and further evidence is needed for a comprehensive explanation. Our preliminary observations found distinctive oily droplet-like substances in astringent seeds under a microscope when they were subjected to forceps pressure, suggesting a difference in lipid metabolism may be present in astringent seeds. Here, the first lipidomics combined with metabolomics analysis was performed on astringent seeds and normal seeds to explore the comprehensive attributes and metabolic alteration in astringent seeds in Chinese fir.

Here, a total of 744 metabolites were detected in the metabolomic analysis across all sampled seeds, with 489 metabolites identified as significantly differentially accumulated (Table S1), indicating obvious metabolic variations in astringent seeds compared to normal seeds. Consistent with previous work, most DAMs classified in categories including saccharides and alcohols, organic acids, amino acids and their derivatives and nucleotides and their derivatives were significant reduced in astringent seeds, and were mainly enriched in essential metabolic pathways such as carbon metabolism (Figure 2), indicative of suppressed elemental cellular activities. Previous research has demonstrated that reductions in specific metabolites, including carbohydrates, amino acids and certain phytohormones, are linked to decreased pollen fertility [23]. Under heat stress conditions, declined in pollen viability and abortion are also linked to decreases in particular metabolites, including carbohydrates and polyamines [34]. Thus, the reduced accumulation of certain essential metabolites might disrupt critical metabolic pathways and compromise the energy and resources necessary for proper embryo development in astringent seeds, thus leading to abortion.

Furthermore, the results of the lipidomic analysis also provide valuable information about the mechanism of astringent seed abortion. In this study, we identified a total of 26 lipid classes and 616 lipid species in two groups of seeds, with 101 lipids identified as significantly differentially accumulated (Table S2). A comparative analysis of the relative abundances of different lipid subclasses detected in the two group of seeds revealed a prevalent decrease in most lipid subclasses in astringent seeds, with most DALs belonging to TG, PA, PC and PE subclasses (Figure 3b,c). Among them, TGs are major storage lipids in seeds; fatty acids resulting from TG breakdown are crucial for membrane biogenesis. TGs function as a significant reserve of fatty acids for carbohydrates and energy production in seed development [35]. Among all the differentially altered lipids, TGs were the most abundant and exhibited the most significant changes (Table 1), suggesting that the reduction in TGs here might be a contributing factor to the decreases in other lipids. In addition, PA is a precursor for the synthesis of various phospholipids that make up cellular membranes, and serves as a key intermediate in lipid-mediated signaling cascades, such as those related to growth, differentiation and stress responses [36]. Additionally, PC and PE are crucial components of cell membranes, contributing to their structural integrity and fluidity [37]. Collectively, these reduced lipid subclasses in astringent seeds were predominantly associated with cell membrane composition and energy storage. The reduction in storage lipids and repressed carbohydrate metabolism might result in a lower energy supply for seed development, while reductions in lipids required for membrane biogenesis and structure maintenance might compromise cell division and expansion, resulting in membrane damage and the observed cell disintegration in astringent seeds [2].

Combining the results from lipidomics and metabolomics can provide more information about astringent seed development. Similar to previous investigations, we also found a significant enhancement in the secondary metabolism, especially flavonoid-related pathways, in astringent seeds (Figure 2e) [8]. The majority of DAMs classified in the tannin and flavonoid categories were more accumulated in astringent seeds (Figure 2c), and almost all of the top 15 DAMs arranged by fold change were also distributed in these two categories (Table 2). Flavonoids are well known for their antioxidant properties and play important roles in protecting plant cells from oxidative stress [38]. The abundance of tannins is another notable characteristic of astringent seeds. Tannins, a group of polyphenolic compounds, are known for their astringent properties and their ability to bind proteins and other biomolecules [39]. Both flavonoids and tannins possess antioxidant properties and can scavenge ROS [40,41]. Additionally, some metabolites such as shikimic acid, quinic acid, proline and GABA were also significantly more accumulated within astringent seeds. Among these DAMs, shikimic acid and quinic acid are important precursors in the biosynthesis of various secondary metabolites, including various antioxidants [42]. Proline could act as an osmoprotectant and an ROS scavenger, helping to stabilize cellular structures and protect cells from ROS-induced damage [43]. Moreover, GABA is a non-protein amino acid and plays important roles in ROS scavenging and stress tolerance in many plants. The application of exogenous GABA could effectively reduce ROS levels and modulate phytohormone cross-talk, thereby enhancing tolerance to multiple stresses [44]. Research by Lin et al. showed that the exogenous application of astringent seed extracts could enhance the adaptability of Chinese fir seedlings to stressful high-aluminum conditions [11]. Here, the substantial accumulation of GABA in astringent seeds may play a role in this process. Taken together, the more accumulated DAMs in astringent seeds were all closely involved in defense against oxidative stress, indicating elevated accumulation of ROS might occur in astringent seeds. Indeed, during the process of astringent seed abortion, the decreased activity of peroxidase enzymes could cause ROS accumulation [5]. Moreover, a comparative lipidomic analysis showed that among the 101 identified DALs, unsaturated lipids constituted 94.6% of the 92 less accumulated DALs (Table S2), resulting in an overall reduction in lipid unsaturation in astringent seeds. Existing evidence indicates that the reduction in lipid unsaturation is closely associated with oxidative stress responses induced by various stresses, such as saline alkali conditions [45], high temperatures and drought [46]. For example, in rice, a lipid profiling analysis revealed a notable reduction in highly unsaturated TG and DG under high-temperature stress [47]. Collectively, the increased accumulation of flavonoids/tannins, proline and GABA and the reduction in lipid unsaturation suggest that the cells in astringent seeds were suffering from ROS-induced oxidative stress.

Hormonal imbalances have also been reported to be associated with astringent seed formation in Chinese fir. Previous investigations have shown that the accumulation of IAA and GA decreased gradually, while the content of ABA increased continuously during the abortion of astringent seeds [4]. A decreased accumulation of IAA and multiple intermediates in the IAA biosynthesis pathway was also revealed by metabolomic analysis of Chinese fir [8]. Our work further observed a significant decrease in IAA accumulation in astringent seeds; however, for the first time, we also identified a significant increase in SA accumulation within astringent seeds in Chinese fir. SA is a plant phytohormone known for its role in plant defense and stress responses. Emerging evidence has revealed that increased SA accumulation is associated with the induction of programmed cell death (PCD) in the developing embryo and endosperm [48]. In Thompson Seedless grapes, elevated SA levels can trigger the upregulation of *VvHDZ28*, which can enhance the expression of downstream PCD-related genes and thereby expedite seed abortion [49]. In addition, increased SA accumulation has also been reported to trigger PCD and be involved in the regulation of stamen abortion in female flowers of tung trees [50]. The occurrence of PCD is common during diverse abortion processes [51,52], and all the phytohormones (IAA, GA, ABA and SA) that undergo abnormal changes in astringent seeds have been reported to be involved in the induction of PCD [53]. For instance, ABA-triggered ROS bursts in rice during anther development play a crucial role in inducing tapetal PCD and heat-stress-induced pollen abortion [52]. Previous anatomical observations of Chinese fir ovules revealed that cell death and disintegration occurred in astringent seeds [2,11]. The significant reduction in most lipid subclasses and elemental metabolites also reflects cellular system disruption or a decrease in viable cells within astringent seeds. These findings serve as further evidence supporting the occurrence of PCD in astringent seeds. Hence, we hypothesize that phytohormone imbalances in astringent seeds might be an important cause of the occurrence of PCD in astringent seeds. Furthermore, a comparative lipidomic analysis showed that only the SoP lipid subclass, belonging to sphingolipids, accumulated more in astringent seeds. Altered sphingolipid levels, including SoP, have been linked to PCD and apoptosis [54]. Among these compounds, sphingosine-1-phosphate (S1P) is a member of the sphingosine phosphate (SoP) subclass of sphingolipids, which is well studied and reported to be involved in the promotion of apoptosis [55,56]. Thus, the more accumulated SoP subclass in astringent seeds might also play a role in the promotion of PCD.

**Figure 4:**
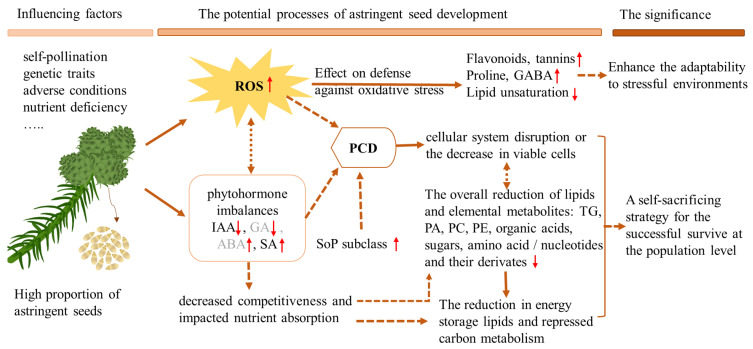
The proposed model underlying the development of astringent seeds in Chinese fir and the potential significance of astringent seed formation. This model highlights the key roles of ROS accumulation and phytohormone imbalances in the induction of astringent seed abortion. ROS, reactive oxygen species; PCD, programmed cell death; IAA, indole-3-acetic acid; GA, gibberellin; ABA, abscisic acid; SA, salicylic acid; SoP, sphingosine phosphate; GABA, γ-aminobutyric acid. The up and down arrows indicate more-accumulated and less-accumulated in astringent seeds, respectively.

Based on existing investigations and the results of this study, we propose a potential model underlying the development of astringent seeds in Chinese fir (Figure 4). The formation of astringent seeds can be triggered by various factors, including self-pollination, differences in genetic traits, adverse conditions and phytohormone or nutrient imbalances [1,3,4,9]. The elevated accumulation of ROS might be a common impact of these factors that triggers astringent seed formation. For example, self-pollination is recognized as one of the predominant factors responsible for the formation of astringent seeds [10], and the influence of differences in genetic traits on astringent seed formation might be linked to the occurrence of highly homozygous states due to inbreeding or self-pollination in certain genotypes [1,12]. Self-pollination-triggered high levels of ROS in the stigma are well reported in the regulation of self-incompatibility in diverse plants [57]. In addition, increased ROS accumulation is a also common phenomenon when plants suffer adverse conditions or have nutrient deficiencies [58]. During the abortion of astringent seeds, the decreased activity of peroxidase enzymes could also facilitate ROS accumulation [5]. The increase in ROS levels could trigger oxidative stress defense mechanisms within viable cells in astringent seeds, including higher accumulation of antioxidant metabolites such as flavonoids, tannins, GABA and proline, as well as the reduction in lipid unsaturation. On the other hand, phytohormone imbalance might be another consequence of diverse factors that triggers the abortion of astringent seeds. We found that SA was more accumulated while IAA was less accumulated in astringent seeds, while increased ABA accumulation and decreased IAA and GA levels were also reported in previous work [4,8]. ROS bursts, abnormal accumulation of these phytohormones, and more accumulated SoP lipid subclass in astringent seeds were all closely involved in the promotion of PCD. The occurrence of PCD in astringent seeds was evidenced by the observed cell disintegration. The overall reduction in most lipid subclasses and elemental metabolites also reflects disruption in the cellular system or a reduction in viable cells within astringent seeds. Taken together, it is reasonable to propose that the accumulation of ROS and the occurrence of PCD are critical factors underlying various physiological and biochemical changes observed in astringent seeds. Increased levels of SA and SoP in astringent seeds may play important roles in the promotion of PCD. Furthermore, phytohormone imbalances could diminish the competitive ability of astringent seeds and reduce nutrient absorption. Considering the coordinated changes in hormones, the elevation in SoP levels, the reduction in antioxidant enzyme activity [5] and the reduced storage of lipids and carbon metabolism in astringent seeds, we propose that astringent seed formation might represent a selective self-sacrifice strategy employed by Chinese fir under adverse conditions or in the presence of limited resources. This strategy primarily aims to ensure the successful development of a subset of higher-quality seeds, thus maintaining the continuity of the population. However, the significance and mechanisms of astringent seed formation warrant more comprehensive research. Further investigations are also needed to uncover the exact molecular mechanisms linking differentially accumulated metabolites/lipids to seed abortion and to provide a comprehensive understanding of their roles in the formation of astringent seeds.

## 4. Materials and Methods

### 4.1. Plant Materials and Seed Collection

Cones containing seeds were collected from trees growing in Hualin state-owned forest farms. After the seeds were manually extracted from the cones of Chinese fir, they were divided into three types according to their morphology: normal seeds, astringent seeds and empty seeds. As the empty seeds were obviously smaller and thinner with lighter weights compared to other seeds, they were initially removed. Subsequently, the seed coats were carefully dissected, and the embryos were examined under a microscope to distinguish between astringent seeds (SE) and normal seeds (CK) based on their color, luster and hardness. Seeds collected from corns on the same tree were mixed in one group, and two groups, including astringent seeds and normal seeds, were collected, respectively. Three and six biological replicates were prepared for metabolomic and lipidomic analyses, respectively. Samples were immediately frozen in liquid nitrogen and then stored at −80 °C.

### 4.2. Metabolome Sequencing and Data Analysis

Widely targeted metabolomics was carried out on astringent seeds with normal seeds used as controls. The freeze-dried samples were crushed using a mixer mill (MM 400, Retsch) with a zirconia bead for 1.5 min at 30 Hz, and 100 mg of lyophilized powder was extracted at 4 °C overnight with 1.2 mL of 70% methanol solution. Following centrifugation at 12,000 rpm for 10 min, the extracts were filtered (SCAA-104, 0.22 μm pore size; ANPEL, Shanghai, China, http://www.anpel.com.cn/ (accessed on 1 September 2023)) before UPLC-MS/MS analysis. The extraction and detection of metabolites in Chinese fir seed samples were conducted by Wuhan Metware Biotechnology Co., Ltd. (Wuhan, China). The qualitative analysis of metabolites was performed using their self-build database MWDB (Metware database). Subsequently, metabolites data analyses were conducted using Analyst 1.63 software (Sciex, Toronto, ON, Canada). Unsupervised PCA and multi-supervised orthogonal partial least-squares-discriminant analysis (OPLS-DA) were conducted using the r base package and MetaboAnalystrR package, respectively [59]. Metabolites with variable importance in projection values (VIP) > 1 and absolute Log2FC (fold change) ≥ 1 were considered as significantly differentially accumulated metabolites (DAMs). The VIP values were generated from OPLS-DA analysis results. All detected metabolites were annotated using the KEGG Compound database and then mapped to the KEGG Pathway database https://www.genome.jp/kegg/ (accessed on 1 September 2023). Pathways with mapped DAMs were then input into MSEA (metabolite sets enrichment analysis), and a hypergeometric test was applied to calculate *p*-values for significance determination.

### 4.3. Lipidome Sequencing and Data Analysis

Lipids were extracted according to the methyl tert-butyl ether (MTBE) method. Briefly, samples were homogenized with 200 uL water and 240 μL methanol. Then, 800 μL of MTBE was added and the mixture was ultrasonicated for 20 min at 4 °C and then left to rest for 30 min at room temperature. The solution was centrifuged at 14,000× *g* for 15 min at 10 °C and the upper organic solvent layer was obtained and dried under nitrogen. The lipid extracts were re-dissolved in 200 μL 90% isopropanol/acetonitrile and used for LC-MS/MS analysis. The extraction and detection of lipids of Chinese fir seed samples were conducted by Shanghai Applied Protein Technology Co., Ltd. (Shanghai, China). Peak identification, peak extraction and lipid identification (secondary identification) were processed using LipidSearch with the following main parameter settings: precursor tolerance: 5 ppm, product tolerance: 5 ppm, product ion threshold: 5%. Untargeted lipidomic data were processed using LipidSearch version 4.2. Similar to the metabolomic analysis, OPLS-DA was applied to filter and classify irrelevant noise to improve the analytical ability and validity of the model. Lipids with VIP values greater than 1.0 and *p*-values of less than 0.05 were identified as differentially accumulated.

## 5. Conclusions

The prevalence of astringent seeds in Chinese fir significantly hampers the yield and quality of elite seeds, resulting in substantial economic losses. In this work, the first combined lipidomic and metabolomic analysis was performed to gain more insights into the characteristics of astringent seeds. Our results revealed the presence of 744 metabolites and 616 lipids across 26 subclasses. Among these, we identified 489 differential metabolites and 101 differential lipids in astringent seeds. In astringent seeds, there was a notable reduction in the accumulation of elemental metabolites, including amino acids and their derivatives, saccharides and alcohols, organic acids, as well as nucleotides and their derivatives. Additionally, most lipid subclasses were mainly associated with energy storage (TG and DG) and cell membrane composition (PA, PC, PE) and exhibited significant reductions. These changes disrupted critical metabolic pathways, compromised membrane integrity and biogenesis and diminished the energy supply necessary for cell development in astringent seeds, consequently restricting the proper growth of seeds. Conversely, most flavonoids and tannins, GABA, proline and only one SoP lipid subclass were significantly more accumulated in astringent seeds, along with a notable reduction in lipid unsaturation, suggesting that cells in astringent seeds might be experiencing ROS-induced oxidative stress. Furthermore, less accumulation of IAA and more accumulation of SA were also identified in astringent seeds. Taken together, astringent seeds exhibited significant alterations in phytohormones, lipids and various metabolites. The substantial decreases in most lipid subclasses and elemental metabolites reflect a disruption in the cellular system or the occurrence of cell death, causing a reduction in viable cells within astringent seeds. Moreover, the increased levels of SA and SoP were proposed to be closely involved in the promotion of PCD, highlighting the crucial role of PCD in astringent seed development. Collectively, these findings contribute to a deeper understanding of astringent seeds in Chinese fir.

## Figures and Tables

**Table 1:** Alterations in the relative abundance of lipid subclasses in astringent seeds (SE) compared with normal seeds (CK).

Subclass	Full Name	Lipid Types	Mean_SE	Mean_CK	log2FC	*p*-Value	Changes
TG	Triglyceride	Glycerolipids	2,418,466,213.00	398,000,000,000.00	−7.36	0.000	less
SiE	Sitosterol ester	SterolEsters	63,066.94	3,338,359.46	−5.73	0.000	less
DG	Diglyceride	Glycerolipids	49,875,524.58	1,829,902,611.00	−5.20	0.000	less
PG	Phosphatidylglycerol	Glycerophospholipids	88,987,548.34	2,634,724,739.00	−4.89	0.000	less
AGlcSiE	Acylglcsitosterol ester	SterolEsters	8,772,664.30	111,780,732.70	−3.67	0.000	less
LPE	Lysophosphatidylethanolamine	Glycerophospholipids	2,533,033.93	25,965,487.90	−3.36	0.000	less
Co	Coenzyme	Others	4,810,648.04	45,922,404.57	−3.25	0.000	less
LPC	Lysophosphatidylcholine	Glycerophospholipids	12,033,231.72	79,404,329.43	−2.72	0.000	less
SQDG	Sulfoquinovosyldiacylglycerol	Glyceroglycolipids	4,349,611.59	25,803,596.25	−2.57	0.000	less
PA	Phosphatidic acid	Glycerophospholipids	1,000,871,191.00	5,444,599,004.00	−2.44	0.000	less
MGDG	Monogalactosyldiacylglycerol	Glyceroglycolipids	98,227,212.47	461,038,998.00	−2.23	0.000	less
PI	Phosphatidylinositol	Glycerophospholipids	328,142,065.30	1,296,038,926.00	−1.98	0.000	less
DGDG	Digalactosyldiacylglycerol	Glyceroglycolipids	69,555,932.89	252,982,297.10	−1.86	0.000	less
PS	Phosphatidylserine	Glycerophospholipids	106,284,549.40	359,065,750.00	−1.76	0.000	less
PC	Phosphatidylcholine	Glycerophospholipids	2,090,068,304.00	6,189,833,094.00	−1.57	0.000	less
PE	Phosphatidylethanolamine	Glycerophospholipids	703,590,741.40	1,819,163,636.00	−1.37	0.000	less
phSM	Sphingomyelin (phytosphingosine)	Sphingolipids	15,154,103.72	37,533,428.50	−1.31	0.000	less
CerG1	Simple Glc series	Sphingolipids	105,905,712.10	249,572,929.00	−1.24	0.000	less
CL	Cardiolipin	Glycerophospholipids	4,877,980.67	9,895,607.47	−1.02	0.003	less
SoP	Sphingosine phosphate	Sphingolipids	9,236,615.28	4,034,788.16	1.19	0.004	more
LPA	Lysophosphatidic acid	Glycerophospholipids	874,176.31	2,832,812.07	−1.70	0.073	not sig
LPI	Lysophosphatidylinositol	Glycerophospholipids	1,780,832.34	5,315,378.73	−1.58	0.169	not sig
Cer	Ceramides	Sphingolipids	1,341,801,269.00	1,882,630,305.00	−0.49	0.002	not sig
So	Sphingosine	Sphingolipids	122,697,762.80	132,845,014.30	−0.11	0.330	not sig
DGMG	Digalactosylmonoacylglycerol	Glyceroglycolipids	789,186.10	803,985.60	−0.03	0.957	not sig
LPG	lysophosphatidylglycerol	Glycerophospholipids	769,702.94	692,126.93	0.15	0.765	not sig

**Table 2:** The top 15 more accumulated DAMs in astringent seeds.

Formula	Compounds	Class I	Class II	VIP	Log2FC
C_45_H_38_O_19_	Gallocatechin-catechin-catechin	Tannins	Proanthocyanidins	1.07	20.27
C_45_H_38_O_18_	Procyanidin C1	Tannins	Proanthocyanidins	1.07	20.05
C_21_H_24_O_11_	Sieboldin	Flavonoids	Chalcones	1.07	19.17
C_45_H_38_O_18_	Arecatannin C1	Tannins	Tannin	1.07	18.80
C_45_H_38_O_20_	Gallocatechin-gallocatechin-catechin	Tannins	Proanthocyanidins	1.07	18.79
C_27_H_30_O_15_	5,7-Dihydroxy-4-methoxyflavone-3-O-xylose-(1-6)-glucose	Flavonoids	Flavonoid	1.07	17.81
C_60_H_50_O_24_	Cinnamtannin B2	Tannins	Tannin	1.07	17.74
C_15_H_12_O_8_	Dihydromyricetin (Ampelopsin)	Flavonoids	Dihydroflavonol	1.07	17.67
C_21_H_20_O_11_	Luteolin-7-O-glucoside (Cynaroside)	Flavonoids	Flavonoid	1.07	17.63
C_22_H_22_O_13_	Patuletin-3-O-glucoside	Flavonoids	Flavonoid	1.07	17.44
C_60_H_50_O_24_	Arecatannin A1	Tannins	Tannin	1.07	17.31
C_7_H_6_O_4_	2,4-Dihydroxybenzoic acid	Phenolic acids	Phenolic acids	1.07	17.31
C_60_H_50_O_24_	Arecatannin A2	Tannins	Tannin	1.07	17.27
C_22_H_22_O_13_	Laricitrin-3-O-glucoside	Flavonoids	Flavonoid	1.07	17.25
C_7_H_6_O_4_	3,4-Dihydroxybenzoic acid (Protocatechuic acid)	Phenolic acids	Phenolic acids	1.07	17.23

## Data Availability

The data presented in this study are available in the article, Supplementary Materials and online repositories at https://www.ebi.ac.uk/metabolights/MTBLS8712.

## Supplementary Materials

The supporting information can be downloaded at: https://www.mdpi.com/article/10.3390/ijms242015103/s1.
